# Impact of sucroferric oxyhydroxide on the oral and intestinal microbiome in hemodialysis patients

**DOI:** 10.1038/s41598-022-13552-z

**Published:** 2022-06-10

**Authors:** Mohamed M. H. Abdelbary, Christoph Kuppe, Sareh Said-Yekta Michael, Thilo Krüger, Jürgen Floege, Georg Conrads

**Affiliations:** 1 Division of Oral Microbiology and Immunology, Department of Operative Dentistry, Periodontology and Preventive Dentistry, Rheinisch-Westfälische Technische Hochschule (RWTH) University Hospital of Aachen, Pauwelsstr. 30, 52057 Aachen, Germany; 2 Department of Nephrology and Clinical Immunology, Rheinisch-Westfälische Technische Hochschule (RWTH) University Hospital, Aachen, Germany; 3 Department of Operative Dentistry, Periodontology and Preventive Dentistry, Rheinisch-Westfälische Technische Hochschule (RWTH) University Hospital, Aachen, Germany; 4 DaVita Clinical Research GmbH, Geilenkirchen, Germany

**Keywords:** Medicinal chemistry, Gastroenterology, Nephrology, Biofilms, Clinical microbiology, Microbial communities, Clinical trial design

## Abstract

Hyperphosphatemia is a consequence of chronic kidney disease associated with mineral/bone impairment, increased cardiovascular events and mortality. Therapeutically, most dialysis patients have to take phosphate binders. Here, we investigated effects of the Fe(3+)-based phosphate binder sucroferric oxyhydroxide (SFOH) on the oral and gastrointestinal microbiome of 11 hemodialysis patients. Saliva, dental plaque and stool were collected at baseline, one and four weeks of SFOH intake and subjected to 16S rRNA gene (V3-V4 region) directed Illumina MiSeq-based analysis. Total Fe, Fe(2+) and Fe(3+) were determined in stool and saliva. Overall, the microbiome did not change significantly. However, some patient-, sample- and taxon-specific differences were noted, which allowed patients to be divided into those with a shift in their microbiome (6/11) and those without a shift (5/11). Total Fe and Fe(2+) were highest after one week of SFOH, particularly in patients who exhibited a shift in microbiome composition. Eight bacterial taxa showed significant unidirectional changes during treatment. In-depth microbiome analysis revealed that taxa that significantly benefited from iron plethora had no iron-binding siderophores or alternatives, which was in contrast to taxa that significantly declined under iron plethora. Patients with microbiome-shift were significantly younger and had higher serum phosphate concentrations. In conclusion, this study sheds light on the impact of iron on the microbiome of hemodialysis patients.

## Introduction

Hyperphosphatemia is an almost inevitable consequence of chronic kidney disease (CKD), responsible for mineral and bone disorders and associated with increased cardiovascular events and mortality^1^. Therapeutically, most dialysis patients have to take phosphate binders, but this contributes centrally to a high daily pill burden. A phosphate binder with a low pill burden and good tolerability is sucroferric oxyhydroxide (SFOH, Velphoro®), a calcium-free polynuclear Fe(3+)-compound. It is formulated as flavoured chewable tablets (each containing 500 mg of iron) that begin to disintegrate in the mouth and bind phosphate. This process continues in the gastrointestinal (GI) tract across the whole physiologically relevant pH range^2^. Phase I studies demonstrated good patient tolerance and a minimal GI iron absorption^3,4^. A phase II study found that 5–12.5 g SFOH—equivalent to 1–2.5 g Fe(3+)—per day significantly reduced serum phosphorus in hemodialysis patients^5^. A phase III study of our group demonstrated the efficacy, safety, adherence, and low rate of serious adverse events of SFOH^1^. However, a common adverse event is diarrhea (described as ‘loose’ or ‘soft’ stools), but which is generally mild, transient, early in onset, and leading to only few discontinuations.

Little is known about the impact on the human microbiome during SFOH administration, in particular in view of its ferric iron Fe(3+) component, which is generally regarded as a bacterial growth factor, especially if reduced to ferrous iron Fe(2+). Recently, Iguchi et al.^6^ found no significant change in bacterial diversity investigating all taxonomic levels, but potential uremic toxins such as serum indoxyl sulfate and *p*-cresyl sulfate were significantly elevated in the test group. Merino-Ribas et al.^7^ confirmed the long-term stability of the gut microbiome on SFOH in a pilot study based on seven patients and another five patients switching to calcium acetate as phosphate binder. Both studies were limited to the intestinal microbiome, whereas major effects would be expected to occur in the oral cavity given the rapid disintegration of SFOH in saliva. Here, we investigated the effects of SFOH administration on the oral and gastrointestinal microbiome in a phase IV clinical trial. Our null-hypothesis was that the microbiome does not change during SFOH-administration.

## Results

### Characteristics of the study population including serum parameters

**Figure 1:**
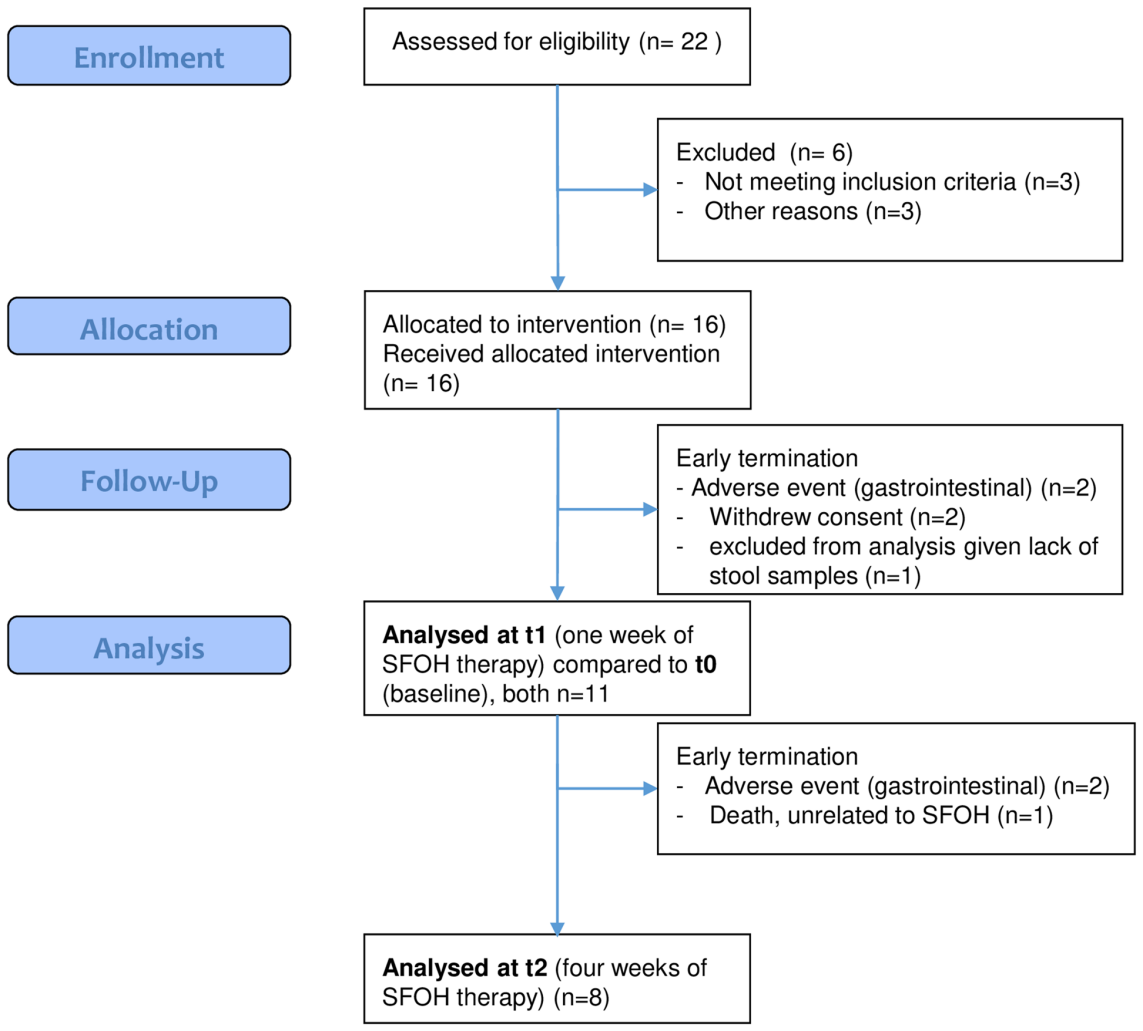
CONSORT flow diagram of the study.

Out of 16 allocated patients, 5 individuals terminated study participation and SFOH-medication early because of gastrointestinal adverse events (2), withdrawal of consent (2) or lack of a stool sample (1); therefore, 11 patients were included in the final analysis. Details about enrollment, allocation, follow-up, and analysis are given by the CONSORT flow chart (Fig. 1). Patients received 500 mg/day SFOH with each meal, in place of other phosphate binders. The dosage of other medicines, such as blood pressure medications, was not changed during the study period. Patient characteristics and baseline clinical chemistry parameters for individual patients (Table 1) and over all patients (Supplementary Table ST1) are given.

**Table 1:** Clinical baseline characteristics of each patient.

Patient	Age (y)	Height (cm)	BMI (kg/m^2)^	DW (kg)	PPB	Duration dialysis	Cause of ESRD	DM/nonDM	Protein (g/dl)	
A01	44	170	27.7	80.5	SVC	12–24 months	Atypical uremic hemolytic syndrome	non-DM	6	
A02	58	193	31.9	115,5	SVC, CAA	12–24 months	Diabetes type 2	DM	7.2	
A05	83	180	20.4	67	SVC	> 36 months	Nephrosclerosis, hypertension	non-DM	6.4	
A08	79	175	24.5	76.5	SVC	12–24 months	Diabetes type 2	non-DM	5.8	
A09	43	191	24.4	81	SVC, LAC	12–24 months	Glomerulopathy, thin-basement syndrome	non-DM	**n.d**	
A12	64	176	22.6	71.5	CAA, MGC	24–36 months	Nephrosclerosis, hypertension	non-DM	5.9	
A13	77	182	26.0	86	CAA, MGC	36–48 months	Secondary, vascular, aortic aneurysm	non-DM	**n.d**	
A15	66	192	31.5	116	SVC	> 48 months	Diabetes type 2	non-DM	7.1	
A16	56	158	30.8	77	SVC/CAA, MGC	36–48 months	Interstitial nephritis	non-DM	**n.d**	
A17	80	167	21.9	60	SVC	36–48 months	Nephrosclerosis, hypertension	non-DM	6.3	
A19	76	159	24.5	59	SVC	> 48 months	Nephrosclerosis, hypertension	non-DM	5.9	

	Ca (mmol/l)	P (mmol/l)	iCa (mg/dl)	wPTH (pg/ml)	Hb (g/dl)	Fe (ug/dl)	TSAT (%)	Ferritin (ng/ml)	**pH**	**HCO3**
A01	2.01	2.1	1.11	278	10.8	58	18.6	57.6	7.34	22.8
A02	2.11	1.77	1.06	398.8	9.7	34.4	9.5	117.7*	7.43	27.9
A05	2.68	1.7	1.36	265.7	9.6	33.2	13.2	606.2	7.42	27.6
A08	2.19	1.15	1.13	138.2	11.9	74.2	32.5	996.8	7.42	23.9
A09	2.49	2.87	**n.d**	384	10.8	50.5	22	753	**n.d**	**n.d**
A12	2.19	2.9	1.11	188.9	13.3	78.9	20	80	7.35	19.7
A13	2.25	1.58	**n.d**	**n.d**	11.6	66.2	24	1171*	**n.d**	**n.d**
A15	2.32	1.33	1.11	150.8	10.4	98.5	31	584.1	7.35	19.7
A16	2.24	2.08	**n.d**	649	11.1	66.8	36	954	**n.d**	**n.d**
A17	1.94	1.39	0.99	194.9	9.0	33	14.5	481.4	7.41	20.8
A19	1.98	1.77	1,04	228.5	10.8	68	26.1	354.5	7.32	23.9

BMI = body mass index, DW = dry weight on dialysis, ESRD = end-stage renal disease, PPD = primary phosphate binder (SVC = sevelamer carbonate, CAA = calcium acetate, LAC = lanthanum carbonate, MGC = magnesium carbonate), DM = diabetes mellitus, blood serum (or whole blood in case of pH & HCO3) values: P = phosphate, iCa = ionized calcium, Ca = total calcium, wPTH whole PTH, Hb = hemoglobin, Fe = iron, TSAT = transferrin saturation, HCO3 = bicarbonate (mmol/l); n.d. not determined; *patients on parenteral iron, possibly further increasing ferritin (A02, A13).

### Changes in the microbiome across all patients and samples

**Figure 2:**
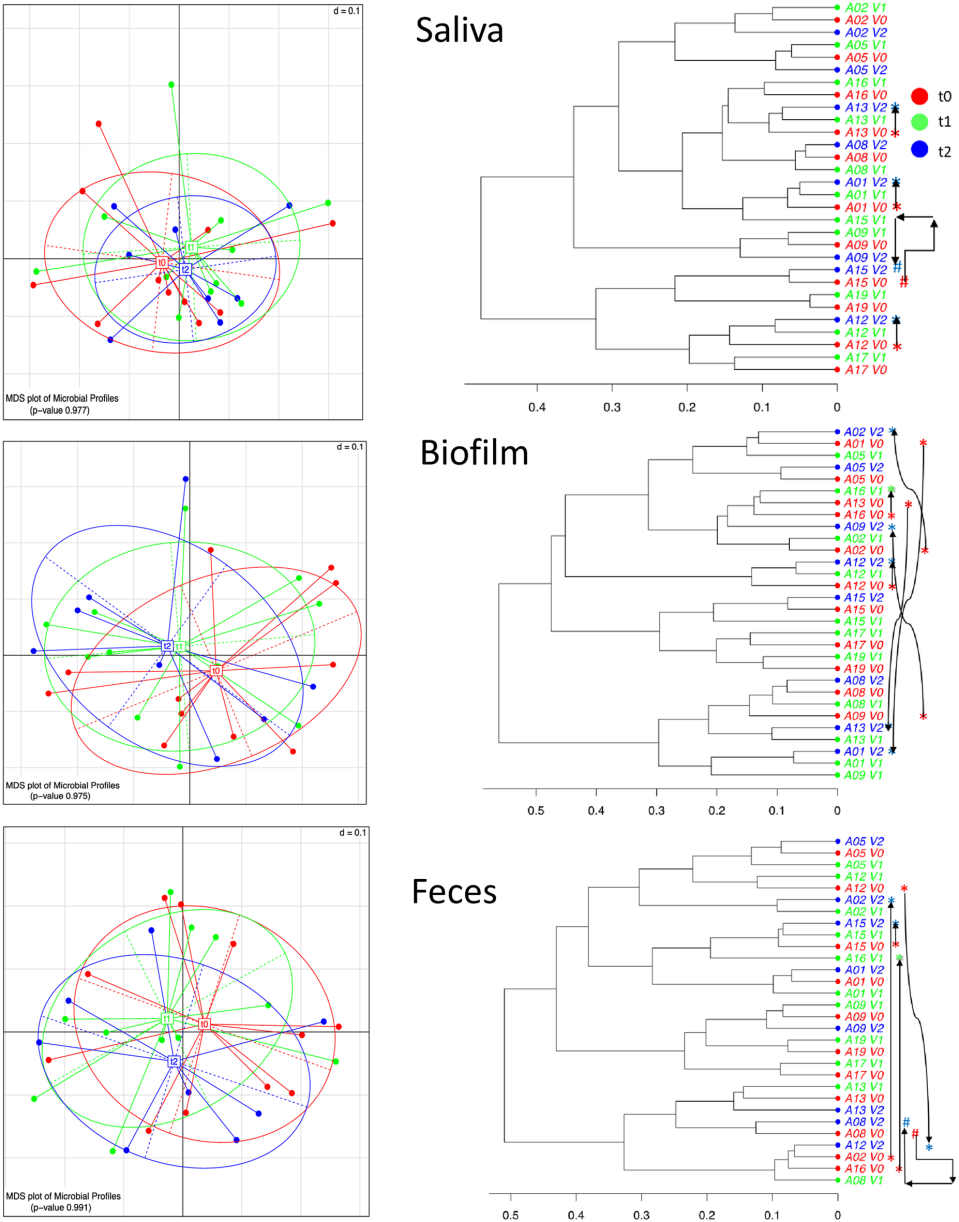
Analysis of β-diversity. Left: multidimensional scaling (MDS): over all taxa, the beta-diversity was not significantly different between the saliva, biofilm, and fecal samples collected at three different time points, t0 (before switch to SFOH), t1 (one week after SFOH switch), and t2 (four weeks after SFOH switch). Right: Cluster analysis and intra-patient dynamical change of microbiome. Changes that appear significant are followed by arrows. Patients with such a shifting microbiome (named shifters) were A01 (saliva, biofilm), A02 (biofilm, feces), A09 (biofilm), A12 (saliva, biofilm, feces), A13 (saliva, biofilm), and A16 (biofilm, feces, with data for t0/1 only). Microbiomes of patients A05, A08, A15, A17, A19 (non-shifters) appeared quite stable, independent of specimen. In a subgroup here, however, two specimens (saliva of A15 and feces of A08) changed from t0 to t1, but t2-microbiome clustered again with t0, following a boomerang move.

We obtained 30 saliva, 30 biofilm and 30 fecal samples, with 11 samples/patient taken at baseline (t0, before switch of medication) and at one week (t1) of SFOH-medication, while 8 samples/patient were taken at four weeks (t2) of SFOH-medication. We achieved an average of 24,650 (saliva), 24,356 (biofilm) and 19,538 (feces) high quality-trimmed sequence reads per sample. Multidimensional scaling analysis (MDS) of β-diversity revealed that most differences between microbiomes were patient and/or specimen dependent. Across all patients, the microbiome did not change significantly after SFOH intake and the null-hypothesis was confirmed. However, two groups of responses to SFOH could be distinguished. The saliva MDS-plot and phylogenetic clustering were the most condensed and the shift in microbiome (from t0 to t1 to t2) was minor and limited to three patients (A01, A12, A13) (Fig. 2, shift is indicated by arrows). In contrast and even after fair sample homogenization, the biofilm MDS-plot and clustering showed a wider range of interpatient microbiome diversities. In particular, the biofilm samples showed a clear shift in microbiome-composition in six patients: A01, A02, A09, A12, A13, and A16 (Fig. 2, right side). The feces MDS-plot and clustering showed a shift in four patients (A02, A12, A15, and A16) (Fig. 2). Summarizing the overall results here, relevant shifts (12 events in total) in the microbiome were found in patients A01 (saliva, biofilm), A02 (biofilm, feces), A09 (biofilm), A12 (saliva, biofilm, feces), A13 (saliva, biofilm), and A16 (biofilm, feces, with data for t0/1 only). We henceforth call these patients *shifters*. In contrast, the microbiomes of patients A05, A08, A15, A17, and A19 (referred to as *non-shifters*) appeared quite stable after SFOH administration and regardless of the type of sample tested. In response to SFOH-uptake, stratification of patients into shifters and non-shifters is a key finding of this pilot study.

### In-depth analysis of changes in the oral and intestinal microbiomes after SFOH administration

**Figure 3:**
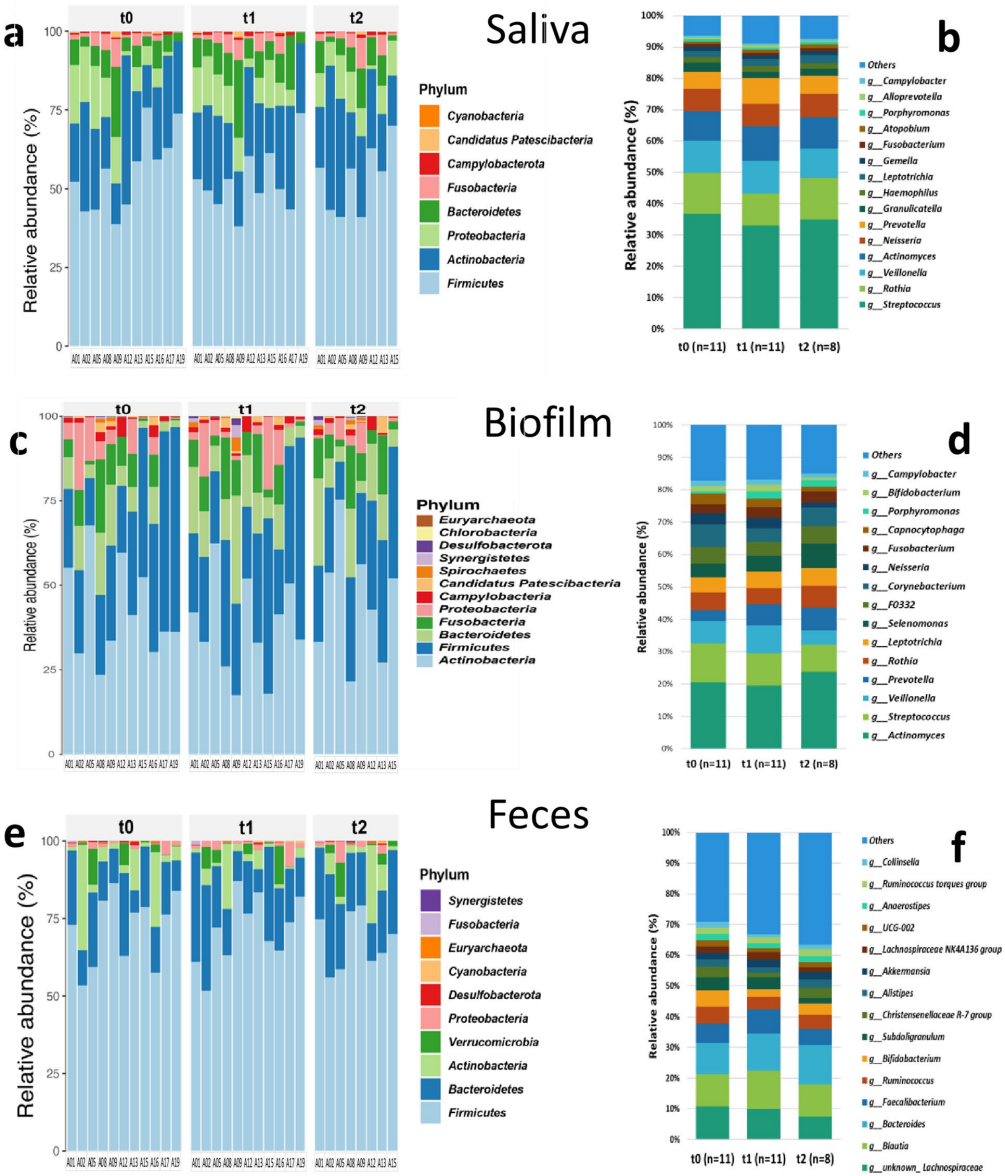
Changes in the oral (**a**–**b** saliva and **c**–**d** teeth-attached biofilm) and intestinal (**e**–**f**) microbiome from baseline (t0) to one (t1) and four (t2) weeks of SFOH administration. Phylum composition is presented on the left (**a**, **c**, **e**) and genera composition on the right side (**b**, **d**, **f**).

The 16S rRNA gene sequencing analysis revealed eight different major phyla in the saliva samples (Fig. 3a), dominated by Firmicutes (t0 median 56.8%, t1 median 50.5%, t2 median 55.5%), Actinobacteria (22.3%, 26.4%, 22.7%), Proteobacteria (7.5%, 10.7%, 10%), Bacteroidetes (6.4%, 8.8%, 8.6%), and Fusobacteria (1.6%, 2.4%, 2.1%). We also identified 15 major genera (Fig. 3b). The biofilm was composed of 12 different major phyla (Fig. 3c). Here, the microbiome was dominated by Actinobacteria (t0 median 36.7%, t1 median 34.3%, t2 median 47%), Firmicutes (28%, 26.3%, 28.1%), Bacteroidetes (7.4%, 12.1%, 11.4%), Fusobacteria (8.5%, 8%, 7.6%), and Proteobacteria (5%, 2.2%, 2%). We again identified 15 major genera (Fig. 3d). The fecal samples presented 10 different major phyla (Fig. 3e). Here, the microbiome was dominated by Firmicutes (t0 median 76.2%, t1 median 72.6%, t2 median 67%), Bacteroidetes (14.9%, 16.6%, 20.8%), Actinobacteria (2.5%, 2.3%, 2.4%), Verrucomicrobia (0.2%, 0.2%, 1.2%), and Proteobacteria (1.1%, 1.1%, 1.1%). The most abundant 15 genera are presented in Fig. 3f. For further details, the reader is directed to the Supplementary Information.

Significant changes after SFOH administration over all bacterial taxa on species, genus, and family level are summarized in Fig. 5. The eight significant unidirectional (t0 to t1 to t2) changes observed in this study were: increase of *Streptococcus salivarius* (p = 0.0439) and oral *Prevotella* (p = 0.0302) in saliva; decrease of *Corynebacterium* (by far dominated by a single species, *C. matruchotii*) (p = 0.0498), *Capnocytophaga* and *Neisseriaceae* (latter both p = 0.0313) in biofilm; and increase of intestinal *Veillonella* sp. and *Ruminococcus torques* group (both p = 0.0351) as well as decrease of *Subdoligranulum* (p = 0.0496) in fecal samples. Therefore, the Fe-dependent metabolism of these eight bacterial taxa is viewed in detail. Of note, *Atopobium parvulum* in saliva, oral *Veillonella* sp. in biofilm and *Eubacterium coprostanoligenes* as well as *Blautia* sp. showed significant changes but were excluded from further analysis because their dynamics were reversible (increase on t1 and decrease on t2 or vice versa) and not unidirectional.

**Figure 4:**
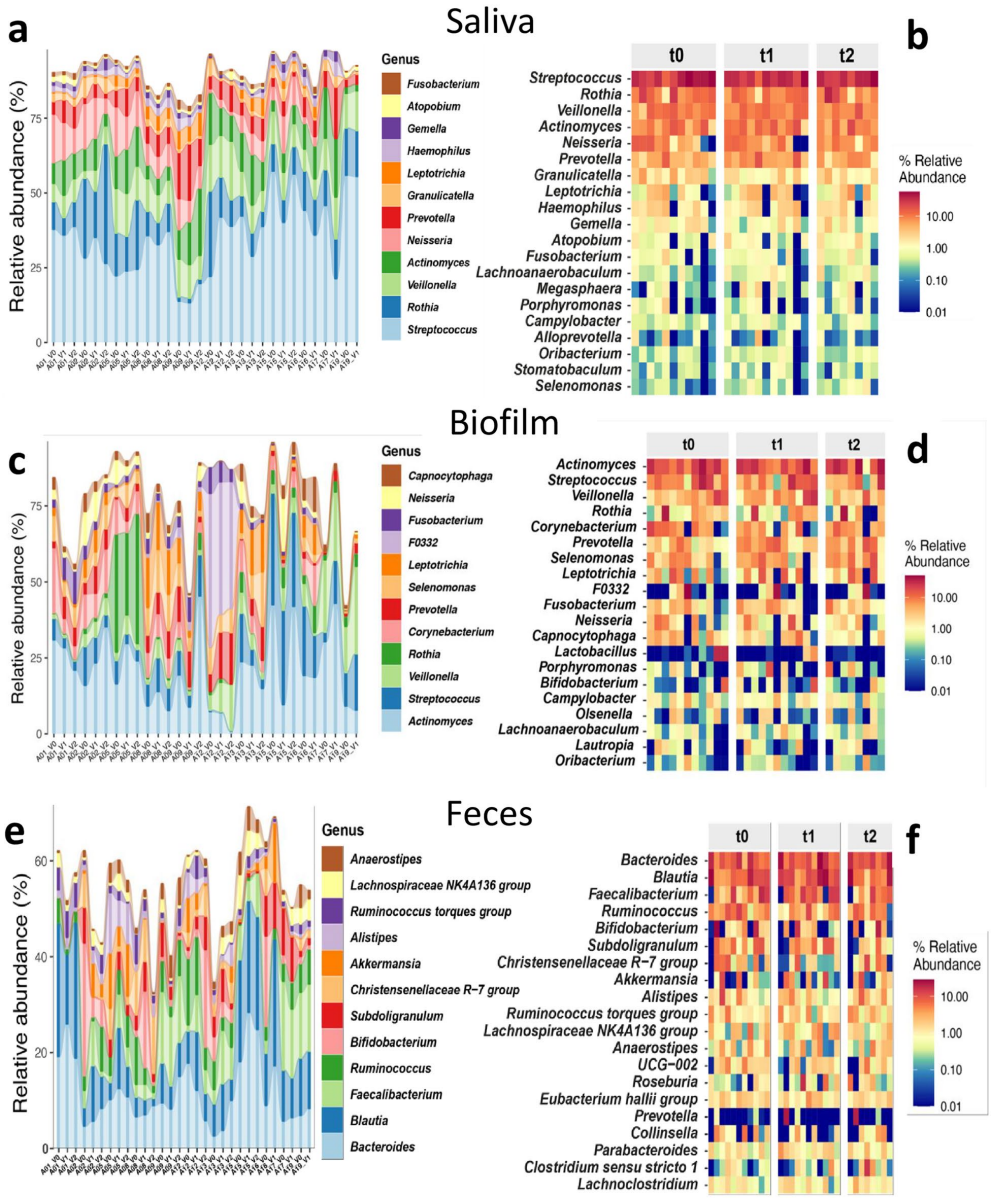
Changes in the oral (**a**–**b** saliva and **c**–**d** teeth-attached biofilm) and intestinal (**e**–**f**) microbiome from baseline (V0, t0) to one (V1, t1) and four (V2, t2) weeks of SFOH administration. The patient-specific dynamic is presented on the left (**a**, **c**, **e**) whereas the relative abundance of each genus is presented on the right side (**b**, **d**, **f**).

**Figure 5:**
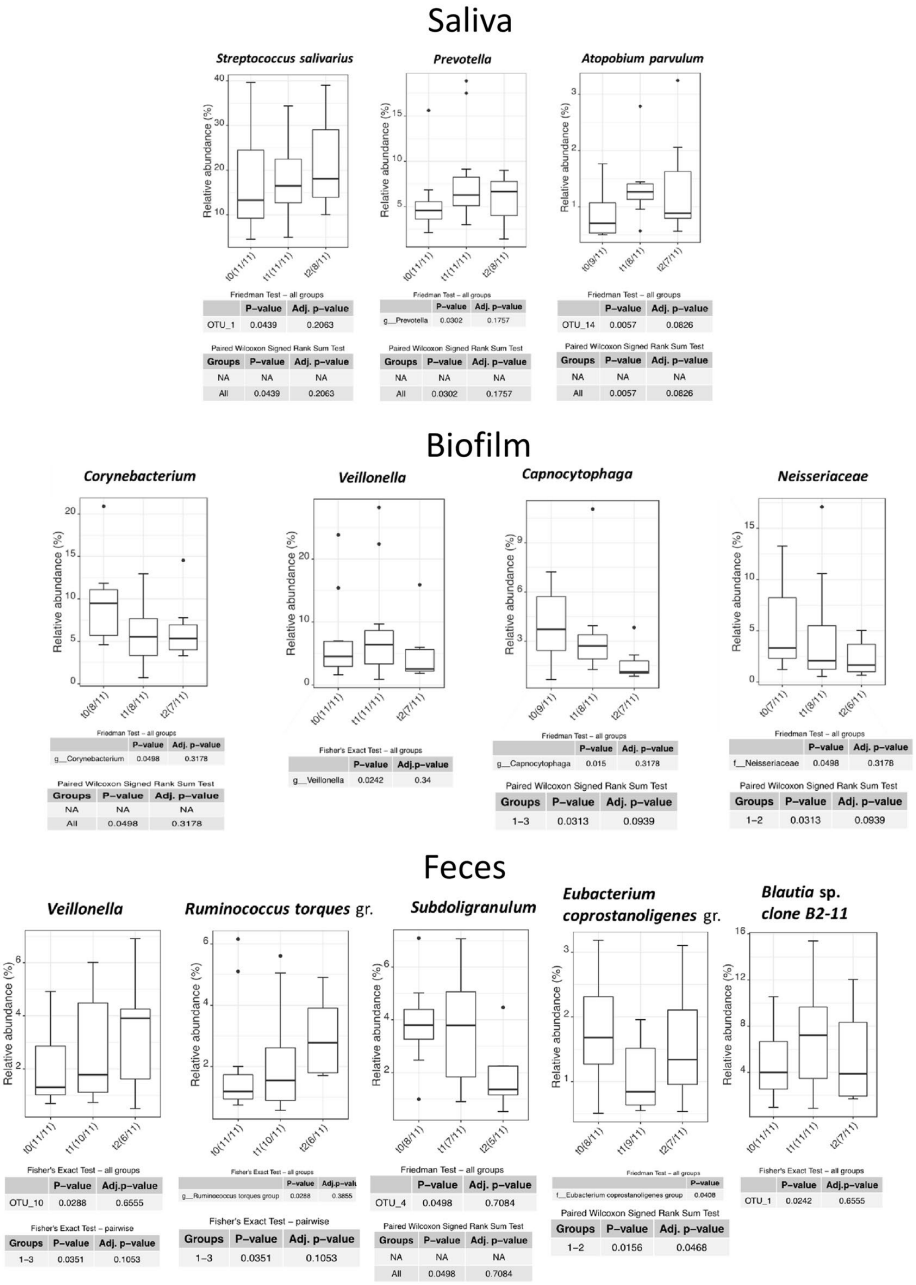
Statistically significant changes in certain taxa of the microbiome in saliva, biofilm, and fecal samples after SFO.

As outlined above, the changes in the microbiome were mainly patient- and sample-specific. The greatest dynamics were observed in the biofilm, followed by fecal samples and saliva. We analysed the most important changes, in total twelve events concentrated in six patients, in depth. The patient-specific dynamic and relative abundances of each genus are shown in Fig. 4. The overall significance of changes in specific taxa (all levels) is shown in Fig. 5. An analysis for each individual patient and event is included in the Supplementary information.

### Changes in pH and iron across all patients

**Figure 6:**
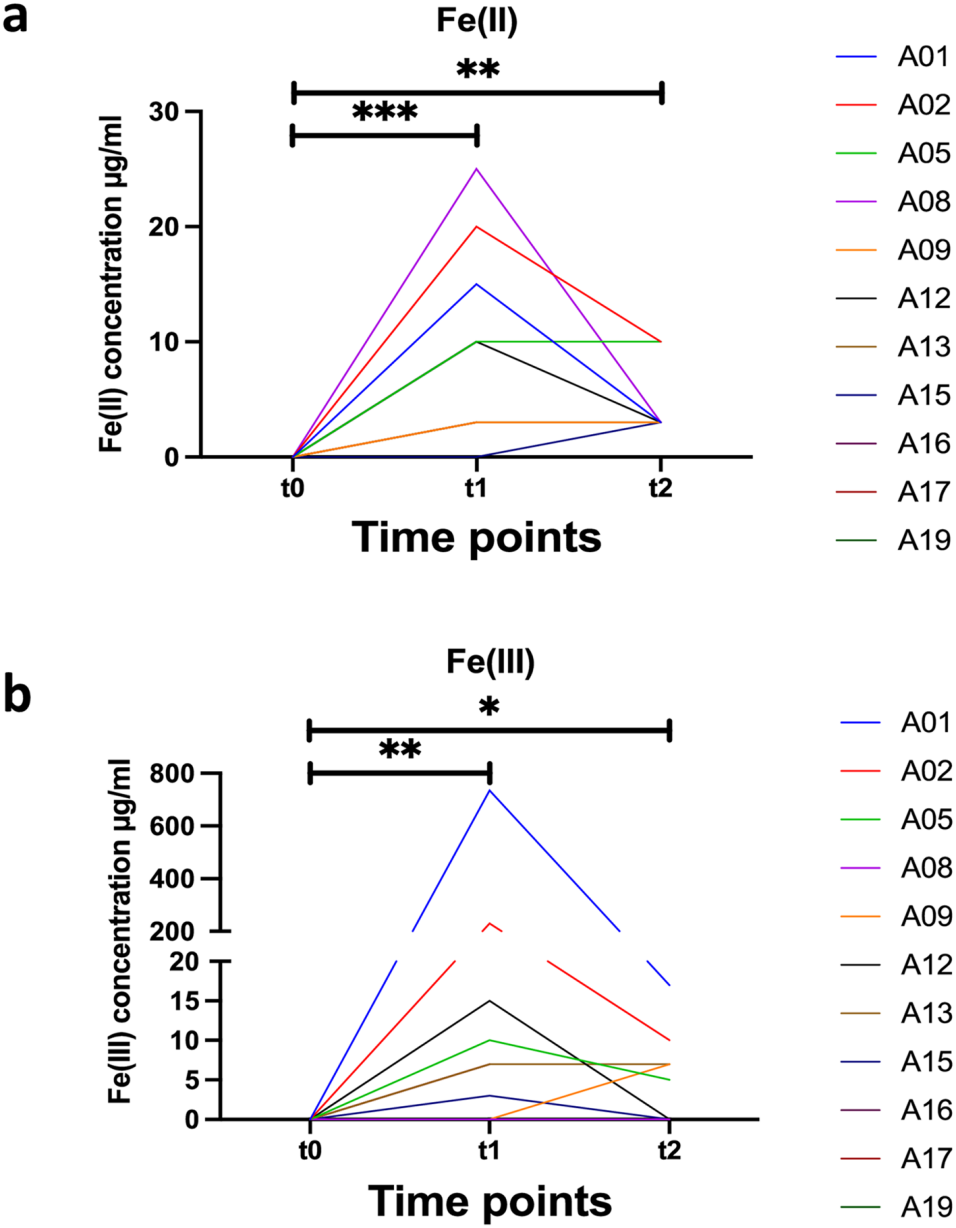
Dynamic of (**a**) Fe(2+) and (**b**) Fe(3+) detected at t0, t1 and t2 in the fecal supernatant of all 11 patients. The statistical analysis was performed using the non-parametric one-way ANOVA (Kruskal–Wallis) as non-pairing due to the missing measurements of patients A16, A17 and A19 at t2. The significance threshold was set at p = 0.05. For Fe(2+) ** = 0.0028 and *** = 0.0003; while for Fe(3+) * = 0.0424 and ** = 0.0085.

**Figure 7:**
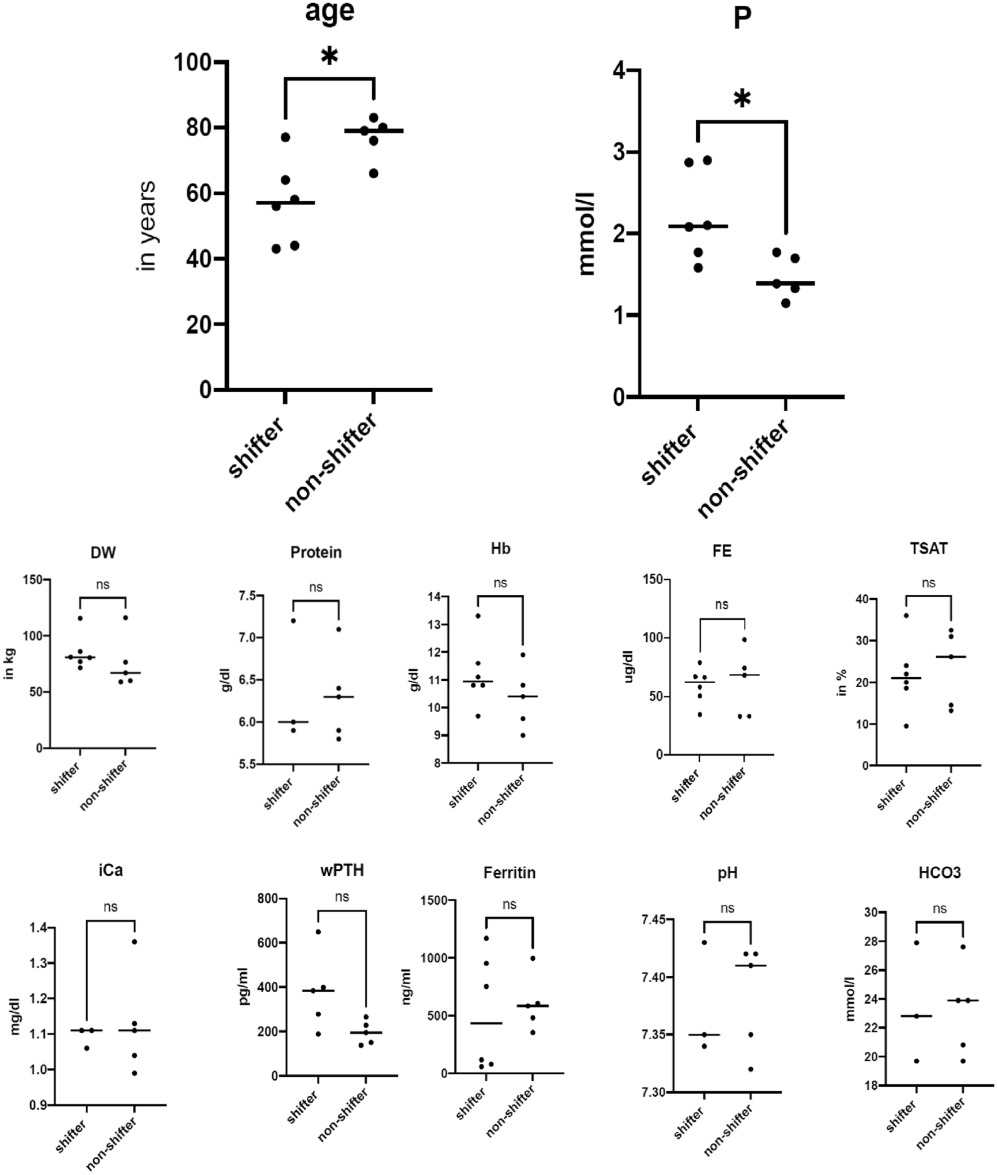
Statistically significant differences of patients’ characteristics and clinical chemistry data (from blood serum or whole blood in case of pH and HCO3), grouped by shifters and non-shifters. DW = dry weight on dialysis, P = phosphate, iCa = ionized calcium, Ca = total calcium, wPTH whole PTH, Hb = hemoglobin, Fe = iron, TSAT = transferrin saturation, HCO3 = bicarbonate (mmol/l). Shifters presented a lower age and higher serum phosphate concentration at baseline. Wilcoxon signed rank sum test. p < 0.05 (*) was considered significant. Differences by height and body mass index were also not significant between both groups.

The redox chemistry of iron is oxygen and pH dependent. Under anaerobic, acidic pH conditions, the reduced bio-accessible ferrous Fe(2+) is preferred. Changes in pH, total Fe (free or chelated) and Fe(2+) were monitored throughout the study in saliva (native and stimulated) and stool (supernatant and sediment). Chewing paraffin gum for stimulation of salivation led to salivary pH increase in most patients. Ferric iron(3+) was calculated by subtracting ferrous Fe(2+) from total iron. Data are presented in Table 2, group-specific changes (delta Δ) in pH and Fe are calculated in Supplementary Table ST2, and significances are shown in Fig. 6. The *non-shifter* group—with a stable microbiome—showed a tendency towards lower mean salivary pH (native 6.5, stimulated 8.0) in comparison to the shifters (native 7.4, stimulated 8.2). Stool supernatant pH was comparable between groups (non-shifters pH 7.3, shifters pH 7.2). It must be noted that salivary pH is to some extend dependent on diet and oral hygiene, both factors for which patients were not adjusted in this study. In contrast the *shifter* group—with a reactive microbiome—had higher total salivary iron concentrations (0.3 µg/ml) at t1, which tended to persist for a further three weeks (t2) in three patients (A02, A12, A13). In contrast, iron was above the detection limit only once in the non-shifters (at t1 in A19). In stool, a much higher total iron content of 174.7 µg/ml was detected in shifters compared to 9.6 µg/ml in non-shifters. Next, we calculated Fe(3+) concentration for each patient and for each time point by subtracting Fe(2+) from total Fe concentration. As expected, total Fe, Fe(2+) and Fe(3+) were below the detection limit at baseline (t0) in all patients and in both saliva and stool samples (supernatant and sediment for the latter). After one week of SFOH intake, a highly significant increase in Fe(2+) and Fe(3+) was observed in the patients' stool supernatant (p = 0.0003 and p = 0.0085, respectively), which then decreased by week 4 (t2, V2, endpoint of the study), but was still significantly higher (p = 0.0028 and p = 0.0424) than at baseline t0 (Fig. 6). In Fig. 7, all patient characteristics and clinical parameters at baseline were analyzed for statistical significance after patients were divided into shifters and non-shifters according to the above microbiome results: shifters were significantly (p < 0.05) younger (mean 57 years vs. 76.8 years, shifters v.s non-shifters) and had higher serum phosphate concentrations at baseline (mean 2.217 mmol/l vs. 1.468 mmol/l).

**Table 2:** Changes in pH and iron content at baseline t0, as well as t1 (one week) and t2 (four weeks) of SFOH administration.

P	S	Saliva				Feces				
		Native	Stimulated			Supernatant			Sediment	
		pH	pH	Total Fe(µg/ml)	Fe(2+) (µg/ml)	pH	Total Fe(µg/ml)	Fe(2+) (µg/ml)	Total Fe(µg/ml)	Fe(2+) (µg/ml)
A01	V0	7.5	8.5	0	0	8	0	0	0	0
A01	V1	7	8.5	0.016	0	7.8	750	15	750	50
A01	V2	8	8.5	0	0	6.8	20	3	30	10
A02	V0	7	8.3	0	0	7.2	0	0	0	0
A02	V1	6	8.3	n.m	2	5.5	250	20	500	50
A02	V2	7	8	n.m	2	7	20	10	60	40
A05	V0	5.5	8	0	0	7.5	0	0	0	0
A05	V1	5.5	8	0.004	0	7.5	20	10	750	500
A05	V2	7	7	0	0	7.5	15	10	750	150
A08	V0	7.5	8	0	0	8	0	0	0	0
A08	V1	7.5	8	0	0	8	25	25	250	250
A08	V2	7.5	8.5	0	0	7.5	3	3	50	50
A09	V0	8.5	9	0	0	7	0	0	0	0
A09	V1	8.5	7	0	0	7	3	3	50	10
A09	V2	6.5	8.5	0	0	7	10	3	50	25
A12	V0	7	7.5	0	0	7	0	0	0	0
A12	V1	7	8	n.m	3	7.5	25	10	250	250
A12	V2	8	8.5	0.16	0	8	3	3	100	100
A13	V0	7	8.5	0	0	7	0	0	0	0
A13	V1	8	8	n.m	1	6.5	10	3	250	50
A13	V2	7	8.5	0.008	0	7.5	10	3	500	25
A15	V0	6.5	8	0	0	7	0	0	0	0
A15	V1	6.5	8.5	0	0	8	3	0	500	50
A15	V2	6.5	8.5	0	0	7	3	3	50	25
A16	V0	8	8.5	0	0	7	0	0	0	0
A16	V1	8	8	0	0	8	10	10	50	25
A16	V2	Drop out								
A17	V0	7	8	0	0	7	0	0	0	0
A17	V1	5.5	8	0	0	5.5	10	3	250	50
A17	V2	Drop out								
A19	V0	7	n.d	0	0	7	0	0	0	0
A19	V1	5.5	7.5	n.m	3	6.5	0	0	3	0
A19	V2	Drop out								

P: patient number, S: time-point (t0, t1, t2) related sample number (V0, V1, V2), n.d. not determined, at V0, the patient did not produce any additional saliva after stimulation. n.m. not measurable: at low concentrations, Fe test kits showed diffuse colors or discrepancies and these cases were excluded as not reliable.

## Discussion

Here, in a phase IV clinical study, we assessed consequences of SFOH administration and intake of 1,500 mg ferric iron per day, for oral/intestinal iron concentrations and stability of the oral and gastrointestinal microbiome. Ferric maltol is an Fe(3+) sugar complex used for oral treatment of anemia in IBD-patients. While oral Fe(2+) supplementations (as sulfate, gluconate, fumarate) are known to alter the microbiome, Fe(3+) maltol had no significant effects on bacterial taxa in mice and human^8^. However, with a therapeutic dosage for iron-supplementation as low as 60 mg per day, the Fe(3+) challenge on the microbiome is much lower compared to the daily 1500 mg by SFOH. With this high dosage, we have measured a highly significant increase in iron in feces (Fig. 6) but which partly discontinued despite the continued medication. This undoubtedly indicates an adaptation of the digestive tract to the high amount of iron ingested, which reduces clinical symptoms. However, from our results we cannot deduce which elements of the iron transport and metabolism system (both on the host and microbial side) are responsible for this adaptation. Collaterally, a significant change in some bacterial taxa was observed which can be divided into two categories.

First, taxa that benefited significantly from iron-plethora in our study (*Streptococcus salivarius*, oral *Prevotella*, intestinal *Veillonella*, and *Ruminococcus torques*) did NOT possess siderophores. Instead, Streptococci are able to use (cadge) ferric ferrochrome, a hydroxamate siderophore produced by other bacteria in the niche, as a source of Fe^9,10^. In a cystic fibrosis co-culture model, it was shown that *Pseudomonas* (a major siderophore producer) controlled the growth of *Streptococcus* (non-producer) as long as iron was limited^11^. In a study looking at the salivary microbiome in individuals with and without caries, it was found that *Streptococcus* sp. as well as most oral *Prevotella* sp. were positively correlated with iron concentration, an evidence for the direct impact of this metal on the growth^12^. Anaerobic bacteria generally do not produce siderophores and may benefit from siderophore and/or iron supplementation. For example, addition of various siderophores to culture media was found to increase the growth of fastidious organism such as oral *Prevotella* (HOT-376) as they consume the Fe-laden siderophores^13^. In the case of anaerobic *Veillonella* and *Ruminococcus torques*, we performed a comprehensive advanced PubMed search, which revealed no evidence of siderophore production. At the molecular level, a review of the GenBank annotations of the *Veillonella atypica* gut isolate MGYG-HGUT-01444 (accession number: CABKSO0000000) or the *Ruminococcus torques* strain AM22-16 (accession number: NZ_QRIH01000001.1) also did not reveal any siderophores. In a recent study investigating the effect of ferric citrate as an alternative to calcium carbonate phosphate binders in eight patients, it was found that members of *Flavonifractor*, *Cronobacter*, and also certain *Ruminococcus* sp. were enriched in patients treated with ferric citrate phosphate binder^14^. All in all, it is plausible that out-competition of siderophore-non-producers is reduced under iron plethora conditions. Comparable to our study, overall, both Iguchi et al.^6^ and Merino-Ribas et al.^7^ found no significant changes in the gut microbiome after SFOH intake. However, Iguchi et al. found a significant increase in the families *Clostridiaceae* (0.6%, 2.6%, p = 0.0264) and *Oscillospiraceae* (0.21%, 0.40%, p = 0.023) and the genus *Oscillibacter* (t0 0.20%, t12weeks 0.44%, p = 0.022). Interestingly, at least the abundance and increasing dynamics of *Oscillibacter* were similar in our study (t0 0.22%, t1week 0.30%, t4weeks 0.42%). After screening the literature and a reference genome (*O. valericigenes* strain Sjm18-20) it can be concluded that this strictly anaerobic genus does not produce any metal binding chelators, again supporting our model. Merino-Ribas et al. concluded that the individual was the most important qualifier and that the long-term treatment with both phosphate binders (calcium acetate and SFOH) did not further modify this diversity^7^. However, without given any details, they found statistically significant differences by ANOSIM (p = 0.002) and PERMANOVA (p = 0.001) between both groups, indicating that influencing factors—with siderophores as candidate—might have been overlooked.

Second, taxa which significantly relapsed under iron-plethora (*Corynebacterium*, *Capnocytophaga*, *Neisseriaceae* in biofilm; *Subdoligranulum* and *Bifidobacterium* in feces) possessed siderophores or alternatives: *Corynebacterium matruchotii* (reference strain ATCC14266 with an accessible genome no. NZ_ACSH02000005), by far the most dominating species in the oral biofilm^15^, produces siderophore biosynthesis proteins of the LucA/LucC family and corresponding Fe(3+)-siderophore ABC transporter permeases. It can therefore be assumed, that under iron plethora the energy loss by continued production of such redundant proteins is disadvantageous for *Corynebacterium*, resulting in out-competing. *Bifidobacterium* showed a trend of decrease in our study worth to discuss. Vazquez-Gutierrez et al. screened six different *Bifidobacterium* species and found in 35 strains (41%) an exhibited high, in 31 strains (36%) an intermediate and in 20 strains (23%) a low, but still measurable siderophore activity^16^. In contrast on the first view, the remaining three taxa discussed in this paragraph do not produce siderophores, but they are known for alternative Fe(3+) capture systems. *Capnocytophaga* possess a polysaccharide utilization locus (PUL) which can act as iron capture system (ICS)^17^. *Neisseria* spp. have also developed alternatives to siderophores. The ferric iron binding protein FbpA is regulated by the level of environmental iron in this genus. Its conservation in all species of pathogenic *Neisseria* has been demonstrated. We searched the genome sequences of apathogenic commensals and discovered an FbpA-encoding gene in *N. lactamica* (strain HMT-649; accession number: NZ_CP031253.1; position: 400,598). Subsequently, the corresponding FASTA gene sequence was used for a blast against Neisseriaceae and it was found that commensal oral *N. mucosa* (HMT-682) and *N. cineria* (HMT-956) also possess FbpA. As mentioned above, there is evidence that FbpA (or other so-called periplasmic binding proteins, PBP) reach the bacterial surface and help to take up Fe(3+) directly from the environment^18^. Finally, data about the iron dependence of *Subdoligranulum* were searched. The taxon, represented by reference species *S. variabile* DSM15176, was described in 2004^19^. The nearest named relative is *Faecalibacterium prausnitzii*. Even staining Gram-negative, this organism is phylogenetically a member of the Gram-positive *Clostridium leptum* supra-generic rRNA cluster. Since its description and inclusion into microbiome-databases and common bioinformatics pipelines, it has been increasingly described as a member of the human fecal flora. By analyzing the annotated genome of the reference strain DSM15176 (accession number: GCA_000157955), we identified a TroA-like metal receptor (GenBank EFB75720.1) that functions as an ABC transporter of iron siderophores, but also for the direct uptake of metal ions, including Fe(3+), which could explain, at least in part, the decrease in abundance in the case of iron plethora.

From the clinical perspective, microbiome shifters were significantly younger compared to non-shifters and had higher serum phosphate concentrations at baseline, while they did not differ significantly in other clinical parameters monitored. Even though the number of participating patients enrolled is rather small (mainly due to the antibiotic therapy as exclusion criterion for most CKD-patients), we believe that the results are valuable. The microbiome of elderly (around 80 y) dialysis patients might have developed a certain robustness and resistance to drastic environmental changes. Many studies address the effects of aging and immune senescence on the human microbiome^20^. Several changes in microbiome structure have been reported to occur with late age, including a reduction in microbiome diversity and increased inter-individual variation. The latter might be the reason why the elderly non-shifters did not cluster in microbiome analyses in our study (Fig. 2, right). Furthermore, in older adults, a reduction in those commensal bacteria that have a beneficial function for the host, such as maintaining mucus production and the integrity of the mucosal barrier, is observed on the one hand, and an emergence of pathobionts on the other^20^. However, the microbiome of elderly dialysis patients is definitely under-investigated^21,22^. The few studies available conclude that the CKD-associated uremia may directly or indirectly affect the composition of the intestinal microbiota, as well as the intestinal barrier^21,22^. The utilization of nutritional therapy (Mediterranean diet, probiotics) may reduce urea levels and restore a physiological microbiome^21^. Our study thus provides the first detailed insight into the composition of the microbiome and its changes in different age groups of CKD. In light of the (indirect) correlation between serum phosphate and gut phosphate^23^, the significance between shifters and non-shifters could have an explanation in the production of siderophores. Phosphate is known to influence the production and activity of siderophores^24^. Phosphate, pH and iron are nearly universal factors that suppress/activate virulence of a wide range of microorganisms relevant to severe intestinal infections and sepsis^25^.

This study has several limitations. First, the number of patients recruited in this pilot study and the heterogeneity in age, gender, oral conditions, and need for parenteral iron-uptake in two patients, make every analysis rather difficult. As a consequence, most taxa lost their significance power after adjusting the p-value for multiple testing. Originally, the study was planned in a case–control design with two groups: hemodialysis patients suffering from hyperphosphatemia (n = 12) and a control group of 12 age- and sex- and oral (dental caries and periodontal) disease status-matched subjects with normal renal function. However, because of juridical and ethical constraint and difficulties in the recruitment of elderly match-partners with a normal renal function, the study was restricted to patients. The exclusion of a healthy comparison group was not essential, but nevertheless complicates the interpretation of the microbiome data. For example, it cannot be ruled out that the observed shifts have other reasons (e.g. important dietary changes) than medication. Second, significant changes in the microbiome were found mainly in four to five taxa that produce siderophores or alternatives and in four to five taxa that do not have this ability. For this reason, we have focused on siderophores here. However, the dynamic of iron through our body and in contact with the microbiome is extremely complicated, and a single explanation can hardly be precise^24^. Third, it is important to note that iron speciation and prediction of solubility through the digestive tract is almost impossible and iron measurement in clinical samples is extremely challenging^24^. For example, the outcome of competition between the many iron ligands is unpredictable as it is pH-and K_a_-dependent. Iron assays may measure sequestered iron that is not normally accessible for bacterial uptake, leading to an overestimation of bioavailability. The reader is referred to some excellent review articles^18,24,26^ to better understand the fate of and battle for iron in the human body and between man and microbiome. Forth, it is important to keep in mind that all microbial traits (such as siderophore activity) are expressed at the species or even strain level, while the microbiome is monitored at a much higher level, usually at the phylum, family or at least genus level.

In conclusion, despite all the limitations described, this study may shed some light on the effects of iron on the microbiome in general and on the microbiome of hemodialysis patients in particular. Although the number of patients recruited over five years between 2016 and 2021 remained small due to the many exclusion criteria, these data could serve as a starting hypothesis to initiate further studies on the dynamics of iron-dependent siderophore producers/consumers. For example, metagenome studies investigating the correlation between the amount of siderophore-encoding genes in the oral and intestinal microbiome and different Fe(3+) and/or Fe(2+) concentrations in saliva, biofilm and fecal samples.

## Methods

### Study design and statements

The study was designed as an open label, pilot study in two centers, the RWTH Aachen University Hospital and the MVZ (Medizinisches Versorgungszentrum) DaVita in Geilenkirchen, Germany. The study was registered with EudraCT (No. 2017–003240-20) and was reviewed and accepted by the ethics committee of the RWTH Aachen University Hospital (No. EK 270/17). Data privacy is following current German standards (DSGVO). Deidentified individual participant data will not be made available. All experiments were performed in accordance with relevant named guidelines and regulations.

### Study population and sample collection

We originally assessed 22 patients of which 11 were finally included and their samples analyzed (CONSORT flow diagram presented as Fig. 1). Age of the included patients ranged from 43 to 83 years (median 65) and the patients had been maintained for at least 1 year on hemodialysis treatment (3 sessions per week of > 4 h). For this pilot study, the sample size calculation and power analysis were difficult because of missing data in humans for the proposed endpoints. Changes in the microbiome of hemodialysis patients and the influence of SFOH (uptake of 1,500 mg ferric iron per day) on both the oral and intestinal microbiome were investigated and clinical data collected during dialysis sessions. The oral and intestinal microbiome before (t0) and one (t1) as well as four (t2) weeks after changing from a non-iron containing phosphate binder (such as sevelamer carbonate, calcium acetate, lanthanum carbonate, magnesium carbonate) to SFOH (Velphoro®, Vifor Fresenius Medical Care Renal Pharma Ltd.)_,_ was monitored. With an estimated dropout rate of 20%, 10 subjects were expected to remain. The following baseline variables were recorded: cause of end-stage renal disease (ESRD), duration of hemodialysis, height and dry weight, body mass index (BMI), and serological parameters. All patients were treated with standard hemodialysis mode. The dialysate calcium (Ca) ion concentration of all participants was 1.25 mEq/l, and this value did not change over the trial period. The inclusion criteria were age ≥ 18 years, hyperphosphatemia, treatment with a non-iron containing phosphate binder at baseline, no or only parenteral iron application (patients A02 & A13), written informed consent prior to study participation, and willingness and ability to follow the procedures outlined in the protocol. The exclusion criteria were age < 18 years, allergy to SFOH, treatment with oral iron supplementation, use of antibiotics within the last two months (important for a stable baseline microbiome but delaying patient recruitment), severe medical events within the last three months, planned surgery for the duration of the sampling, previous major surgery in the gastrointestinal tract, acute/chronic gastrointestinal infections (diarrhea), celiac disease or any other chronic inflammatory bowel disease, smoking, oral candidiasis, oral cancer, pregnancy or lactating, history of haemochromatosis, commitment to an institution by legal or regulatory order, mental or legal incapacity, involvement in a parallel interventional clinical trial, and/or receipt of an investigational drug within 30 days prior to inclusion into this study.

We initially assessed/screened 22 patients with hyperphosphatemia, who agreed to change from other phosphate binders to SFOH and to undergo dental examination, for eligibility (Fig. 1). Of these n = 6 were excluded before any samples were taken. From the remaining 16 patients 2 × 1 ml of saliva, 2 × oral biofilm/dental plaque, 2 × stool, and 2 × blood were taken. Dental plaque samples were collected using a sterile sickle scaler from the occlusal and buccal margin of teeth or existing restorations. A detailed sampling and sample processing protocol is visualized in Supplementary Fig. S1. Important for numbering, t0 (baseline), t1 (one week on SFOH) and t2 (four weeks on SFOH) are time points, whereas V0, V1 and V2 are the standard-labels of the corresponding samples. For initial stool sampling Feces Tubes, with spoon, screw cap, (LxØ) 107 × 25 mm, transparent, and sterile (Sarstedt, Nümbrecht, Germany) were used. Patients were instructed and supervised by a study nurse. For instance, they were instructed to release the stool sample contamination-free on a feces-catcher (www.fecescatcher.com). HydraFlock swabs (Puritan Diagnostics LLC, Maine, USA) were used for optimal sampling and recovery. DNA Stabilizer tubes (DNA/RNA Shield Lysis Tubes Microbe, no. R1103, Zymo Research, Irvine, CA, USA) were used for collection, homogenization, storage, and transport of all samples. The 2 ml-Lysis Tubes are prefilled with 1 ml DNA/RNA Shield Medium and Microbe Bashing Beads (0.6 ml dry volume of mixed 0.5 mm and 0.1 mm beads), ready for chemo-mechanical bead-beating and DNA/RNA isolation. Oral biofilm is an adhesive and cohesive material. By the aid of vortexing in tubes with Bashing-Beads for 2 × 30 s, the biofilm was fairly homogenized. In addition, A (original) and B (backup) aliquots of biofilm were cross-mixed to further homogenize the samples. The pH of unstimulated and stimulated saliva was measured during sampling. For chair-side and laboratory pH-determination pH‑Fix 4.5–10.0, (Macherey–Nagel GmbH & Co. KG, Dueren, Germany) was used. For iron-determination (free and chelator-bound) in stool and saliva, MQuant™ was used (range 3–500 µg/ml, for ferrous Fe(2+)- and [after addition of ascorbic acid reducing all iron to Fe[2+]] for total iron-determination with the same range, Merck KGaA, Darmstadt, Germany). For measurements in stool, a suspension in bidistilled water was prepared, centrifuged and iron measured in supernatant as well as in sediment. In case of a negative result, Iron Sensitive (range 0.004–1.5 µg/ml, N.L. Halanek Chemical Laboratory, Vienna, Austria) was applied but was limited to total iron-measurement in saliva, here expanding the range from 3 down to 0.004 µg/ml.

### Dental examination

The clinical dental/oral examination included the assessment of the periodontal screening index (PSI), DMF-T Score (sum of numbers of decayed, missing and/or filled teeth) and the investigation of gingiva- and tooth discolorations. For assessment of the PSI, the oral cavity was divided into sextants. For each sextant, the highest index was recorded by applying for the following scores: 0 = periodontal health, 1 = gingival bleeding, 2 = calculus and overhanging restorations, 3 = pocket depth (PD) of > 4 but < 6 mm, and 4 = PD > 6 mm. The PSI was investigated with the WHO probe (Aesculap DB767R). Every tooth was probed at four sites (mesio- oral, and disto-oral, disto-vestibular and mesio-vestibular).

### 16S rRNA amplicon sequencing and analysis for microbial profiling

As outlined above, all samples were collected in DNA Stabilizer tubes, which were stored at – 72 °C until processing. After end of patient recruitment and sampling, all specimens were sent on dry ice to the Core Facility Microbiome at the ZIEL Institute for Food & Health, Technical University of Munich, Germany for DNA extraction and 16S rRNA amplicon sequencing. The genomic DNA was extracted from all saliva, biofilm and fecal samples as previously described by Reitmeier et al.^27,28^ using a modified version of the protocol by Godon et al.^29^. After DNA extraction, the V3-V4 regions of the 16S rRNA gene were amplified for all samples using 2-steps PCR with the forward primer 341F-ovh: CCTACGGGNGGCWGCAG and reverse primer 785r-ovh: GACTACHVGGGTATCTAATCC), and subsequently, sequenced on Illumina MiSeq using cartridge v3 with 600 cycles as previously described^28^. Raw sequence reads (2 × 300 base pairs paired-end) were processed for sequence quality check, chimera filtering, and cluster formation using the IMNGS platform^30^, which implements a UPARSE-based Operational Taxonomic Unit (OTU) clustering algorithm at 97% sequence similarity level^31^. In average 24,650, 24,356 and 19,538 merged reads were produced for each saliva, biofilm and fecal sample, respectively. Only OTUs occurring at a relative abundance of 0.25% in at least one sample through the entire saliva, biofilm and fecal sample collection, independently, were kept to avoid the analysis of very rare or false taxa as previously recommended by Reitmeier et al.^32^. For downstream analysis of the produced OTU table, the R pipelines Rhea^33^ and microeco^34^ were used to perform normalization steps, estimation of alpha (within-sample) and beta (between-sample) diversities and generation of the taxonomic classification.

### Statistical analyses

Across paired (over time) input numerical variables, the non-parametric ANOVA for repeated measurement Friedman and Fisher tests were calculated, while for non-paired input numerical variables over selected categorical variables, the non-parametric ANOVA Kruskal–Wallis Rank Sum and Fisher tests were calculated. The pairwise test significance values (p-value) were corrected for multiple testing using the Benjamini–Hochberg method, which is predefined by the Rhea script and is widely used in microbial profiles analysis. For the comparison of clinical data of shifters vs. non-shifters a Wilcoxon signed rank sum test was used. The statistical significance threshold was set at p = 0.05.

## Supplementary Information

Supplementary Information.

## Data Availability

All data generated are provided in Tables 1, 2 and as Supplementary Information. The source data underlying Figs. 2, 3, 4, 5 and all other data are available from the corresponding author on reasonable request. The raw sequence data from this study are made publicly available under NCBI BioProject ID PRJNA813735.
