# Supplementary material for: Impact of sucroferric oxyhydroxide on the oral and intestinal microbiome in hemodialysis patients
Source: Sci Rep. 2022 Jun 10;12:9614. doi: 10.1038/s41598-022-13552-z (PMC9187715; doi:10.1038/s41598-022-13552-z)
Supplement: Supplementary file 1 — Supplementary Information. [file 41598_2022_13552_MOESM1_ESM.pdf]

Supplementary Material

Impact of sucroferric oxyhydroxide on the oral and intestinal microbiome in hemodialysis patients

Mohamed Mostafa Hefny Abdelbary[1$], Christoph Kuppe[2$], Sareh Said-Yekta Michael[3], Thilo Krüger[2,4], Jürgen Flöge[2], and Georg Conrads[1]

1 Division of Oral Microbiology and Immunology; Department of Operative Dentistry, Periodontology and Preventive Dentistry; Rheinisch-Westfälische Technische Hochschule (RWTH) University Hospital; Aachen, Germany

[2] Department of Nephrology and Clinical Immunology, Rheinisch-Westfälische Technische Hochschule (RWTH) University Hospital; Aachen, Germany

[3] Department of Operative Dentistry, Periodontology and Preventive Dentistry; Rheinisch-Westfälische Technische Hochschule (RWTH) University Hospital; Aachen, Germany

[4] DaVita Clinical Research GmbH, Geilenkirchen, Germany

[$]shared authorship

Supplementary Material is provided for three categories

- **I) Supplementary Figures and Tables,**
- **II) Supplementary information about the impact of iron in human and in the microbial world and**
- **III) Supplementary microbiome data (genus level, patient level)**

I) Supplementary Figure and Tables

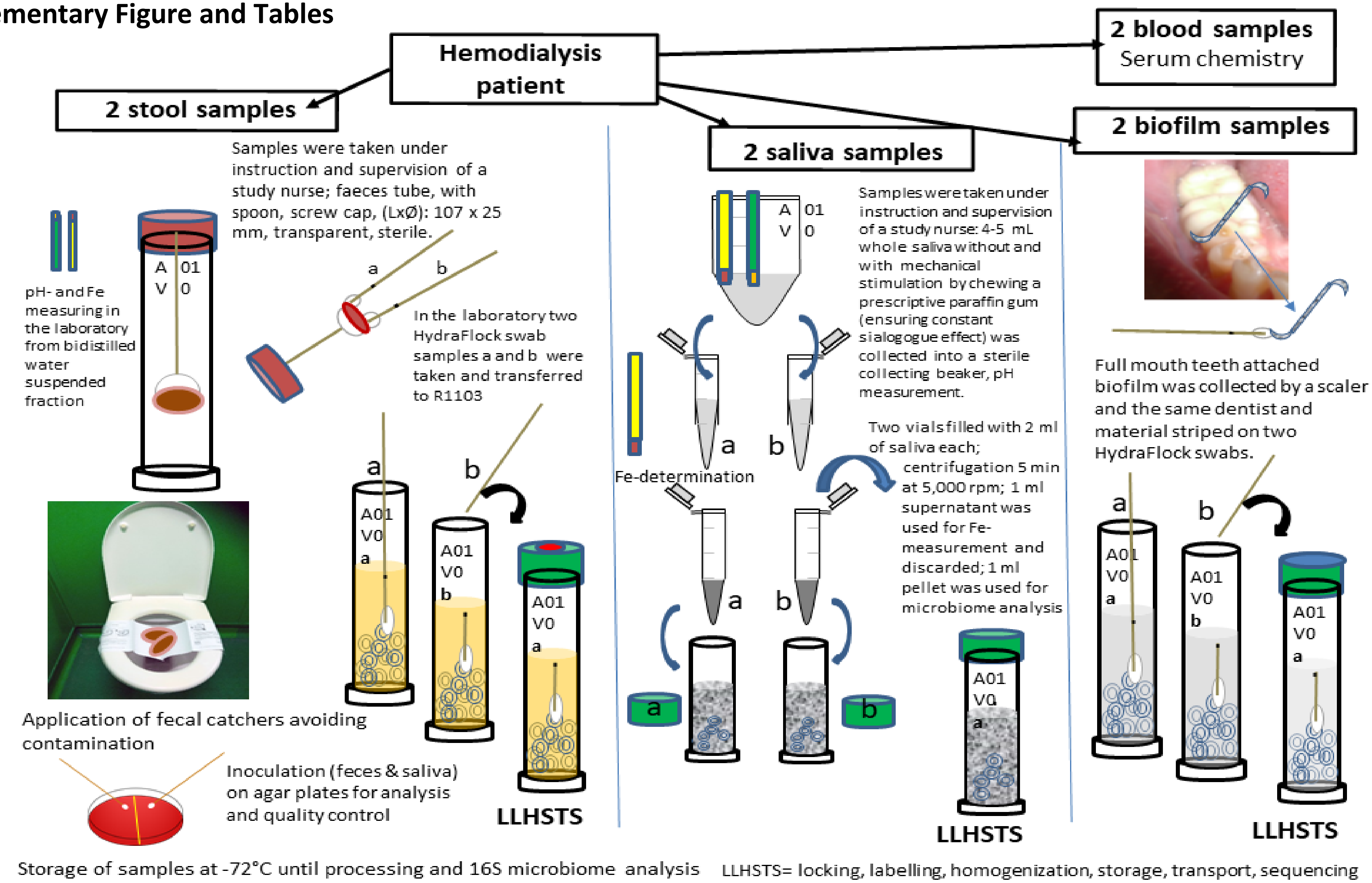

Supplementary Figure S1. Experimental overview with detailed sampling and sample processing protocols.

Supplementary Table ST1. Clinical baseline characteristics over all patients.

variable	N*	Mean	SD	SEM
age (in years)	11	66	14.34	4.323
dry weight (in kg)	11	80.86	19.11	5.763
protein (g/dl)	8	6.325	0.5497	0.1943
P (mmol/l)	11	1.876	0.5771	0.174
iCa (mg/dl)	8	1.109	0.1146	0.04051
Ca (mmol/l)	11	2.218	0.2213	0.06673
wPTH (pg/ml)	10	287.7	154.8	48.96
Hb (g/dl)	11	10.82	1.193	0.3598
Fe (ug/dl)	11	60.15	20.94	6.315
TSAT (in %)	11	22.49	8.432	2.542
ferritin (ng/ml)	11	559.7	385.5	2.542
pH	8	7.38	0.04408	0.01558
HCO3- (mmol/l)	8	23.29	3.225	1.14

*for missing cases compare with Table 1

Supplementary Table ST2. Results of pH- and Fe-measurements as well as changes (delta Δ) from t0 to t1 (before – after SFOH) presented by group: shifters (with a changing microbiome, orange) and non-shifters (with a stable or reversing microbiome, green). The means of pH, ΔpH, Δtotal-Fe, and ΔFe(2+) are also presented.

Shifters (six patients)

Saliva

		pH native	delta V1-V0	pH stimulated	delta V1-V0	total-Fe µg/ml	delta V1-V0	Fe2+ µg/ml	delta V1-V0
A01	V0	7,5	-0,5	8,5	0	0	0,016	0	0
A01	V1	7		8,5		0,016		0	
A01	V2	8		8,5		0		0	
A02	V0	7	-1	8,3	0	0	0,8	0	2
A02	V1	6		8,3		0,8		2	
A02	V2	7		8		0		2	
A09	V0	8,5	0	9	-2	0	0	0	0
A09	V1	8,5		7		0		0	
A09	V2	6,5		8,5		0		0	
A12	V0	7	0	7,5	0,5	0	1	0	3
A12	V1	7		8		1		3	
A12	V2	8		8,5		0,16		0	
A13	V0	7	1	8,5	-0,5	0	0	0	1
A13	V1	8		8		0		1	
A13	V2	7		8,5		0,008		0	
A16	V0	8	0	8,5	-0,5	0	0	0	0
A16	V1	8		8		0		0	
**Mean**		**7,4**	**-0,1**	**8,2**	**-0,4**		**0,3**		**1,0**

Feces

		Supernatant pH	delta V1-V0	total-Fe µg/ml	delta V1-V0	Fe2+ µg/ml	delta V1-V0	Sediment total-Fe µg/ml	delta V1-V0	Fe2+ µg/ml	delta V1-V0
A01	V0	8	-0,2	0	750	0	15	0	750	0	50
A01	V1	7,8		750		15		750		50	
A01	V2	6,8		20		3		30		10	
A02	V0	7,2	-1,7	0	250	0	20	0	500	0	50
A02	V1	5,5		250		20		500		50	
A02	V2	7		20		10		60		40	
A09	V0	7	0	0	3	0	3	0	50	0	10
A09	V1	7		3		3		50		10	
A09	V2	7		10		3		50		25	
A12	V0	7	0,5	0	25	0	10	0	250	0	250
A12	V1	7,5		25		10		250		250	
A12	V2	8		3		3		100		100	
A13	V0	7	-0,5	0	10	0	3	0	250	0	50
A13	V1	6,5		10		3		250		50	
A13	V2	7,5		10		3		500		25	
A16	V0	7	1	0	10	0	10	0	50	0	25
A16	V1	8		10		10		50		25	
		**7,2**	**-0,2**		**174,7**		**10,2**		**308,3**		**72,5**

Non-Shifters (five patients)

Saliva

		pH native	delta V1-V0	pH stimulated	delta V1-V0	total-Fe µg/ml	delta V1-V0	Fe2+ µg/ml	delta V1-V0
A05	V0	5,5	0	8	0	0	0,004	0	0
A05	V1	5,5		8		0,004		0	
A05	V2	7		7		0		0	
A08	V0	7,5	0	8	0	0	0	0	0
A08	V1	7,5		8		0		0	
A08	V2	7,5		8,5		0		0	
A15	V0	6,5	0	8	0,5	0	0	0	0
A15	V1	6,5		8,5		0		0	
A15	V2	6,5		8,5		0		0	
A17	V0	7	-1,5	8	0	0	0	0	0
A17	V1	5,5		8		0		0	
A19	V0	7	-1,5	n.d.	n.d.	0	0	0	0
A19	V1	5,5		7,5		0		3	
**Mean**		**6,5**	**-0,6**	**8,0**	**0,1**		**0,0**		**0,0**

Feces

		pH	delta V1-V0	total-Fe µg/ml	delta V1-V0	Fe2+ µg/ml	delta V1-V0	total-Fe µg/ml	delta V1-V0	Fe2+ µg/ml	delta V1-V0
A05	V0	7,5	0	0	20	0	10	0	750	0	500
A05	V1	7,5		20		10		750		500	
A05	V2	7,5		15		10		750		150	
A08	V0	8	0	0	25	0	25	0	250	0	250
A08	V1	8		25		25		250		250	
A08	V2	7,5		3		3		50		50	
A15	V0	7	1	0	3	0	0	0	500	0	50
A15	V1	8		3		0		500		50	
A15	V2	7		3		3		50		25	
A17	V0	7	-1,5	0	0	0	0	0	0	0	0
A17	V1	5,5		10		3		250		50	
A19	V0	7	0	0	0	0	0	0	0	0	0
A19	V1	6,5		0		0		3		0	
		**7,3**	**-0,1**		**9,6**		**7,0**		**300,0**		**160,0**

II) Supplementary information about the impact of iron in human and in the microbial world and

Iron in human

Iron represents an essential trace element for the proper functioning of all living cells. It occurs in nature as an insoluble Fe(3+) hydroxide polymer. Iron is contained in active centers of many redox enzymes. It plays an integral role in electron transfer, a process where reactive oxygen species (ROS) are generated. Superoxide radicals can cause cell damage and result in apoptosis. Therefore, iron levels in the body need to be tightly controlled. Iron is stored in hepatocytes and macrophages in the liver and spleen. Hepcidin, a hormone produced by the liver, is the main regulator of systemic iron levels (Science review available). Iron requirements are high during infancy, childhood and pregnancy. Ferric iron in the diet is converted to ferrous iron by duodenal cytochrome b. There are two fates of iron according to the body's requirements. Iron not immediately required by the body is stored (absorbed) within **ferritin**. When iron demand is high in the body, it is exported into the circulation via ferroportin1 and ultimately binds to **transferrin and/or lactoferrin**. In the presence of iron supplementation, there is potential to exceed the absorptive capacity (10–20 mg/day) and the unabsorbed iron becomes available to e.g. colonic bacteria, which promotes the growth of some species. During systemic inflammation (Il-1 and -6 as signals) the body reduces iron uptake by increasing hepcidin production (and more is left for gut bacteria) but in IBD (body losses blood) hepcidin levels decrease as anemia and iron-deficiency signals are dominating and more iron is absorbed. Fe(3+) is reduced by bacterial and enterocyte membrane-bound reductases to Fe(2+) before uptake.

Iron in the microbial world

The centrality of iron for bacterial survival and vigor is confirmed by the high level of genomic investment in iron-acquiring mechanisms and by the frequent concentration of such genes in high-pathogenicity islands [1]. Most bacteria can uptake ferrous iron. In the gut, if the duodenal enterocyte absorptive capacity for Fe(2+) of 10-20 mg/day is exceeded, the microbiome will quickly consume the rest. But usually iron is very limited and it needs Fe(3+)-siderophores (small, high affinity iron-chelating compounds) for binding and energy for intracellular reduction to Fe(2+). There are more than 500 known bacterial siderophores. Not all human pathogens (and only a very few commensals) do produce siderophores. Only a narrow number of usually aerobic microorganisms (certain gram-positive Actinobacteria such as *Bifidobacterium*, *Corynebacterium*, *Mycobacterium*, *Nocardia*, *Streptomyces*, and gram-negative Proteobacteria such as *Acinetobacter*, *Bordetella*, *Burkholderia*, *Escherichia*, *Legionella*, *Pseudomonas*, *Serratia*, and *Yersinia*) invest energy, synthesize and secrete the siderophores, which are then taken up as Fe(3+) -complexes via specific ferric iron/Fe-chelator ABC transport systems by them and others ("cadger" which profit without investing energy for siderophore production). Thus, the production of siderophores is an altruistic act as the bacterium that produces them has no guarantee that it will benefit from them. A bacterium that relies on other bacteria to produce siderophores but does not produce them itself is behaving selfishly, as it benefits while other bacteria pay the cost. Gram-negative bacteria concentrate Fe(3+)-siderophores at the cell surface by binding them to very specific high-affinity transport proteins (FepA, FhuA, FecA) and/or concentrate hosts's Fe(3+)-transferrin/lactoferrin (by TbpA, LbpA). Both Fe(3+)-chelators are then transported across the outer membrane into the periplasm. From there, using FbpA shuttles, they cross the periplasmatic gap until they reach TonB and/or ABC transporters (permease proteins) crossing the cytoplasmic membrane into the cytoplasm. Finally, in the cytoplasma, Fe(3+) is released, ATP-dependently reduced to Fe(2+) and incorporated into essential heme and non-heme iron proteins [2-4]. Some bacteria (e.g. *Neisseria*) acquire Fe(2+)-heme from hemoglobin (Hb), myoglobin or directly from the environmental milieu via iron-heme receptor proteins (HpuAB or HmbR). Bacteria grown in anaerobic or in micro-aerophilic environments also need iron but do not seem to produce siderophores. These bacteria have cell surface reductases that facilitate the transport of iron across the outer membrane. It is speculated that FbpA (or other so called periplasmatic binding proteins, PBP) reaches the bacterial surface and may help sampling Fe(3+) directly from the environment (all steps are visualized in Butler 2003 [4]). True for host and microbes: regulation of iron uptake is necessary to maintain optimal non-toxic levels of intracellular iron.

IIIa) Supplementary microbiome data (genus level)

In-depth analysis of changes in the oral and intestinal microbiomes after SFOH administration

The 16S rRNA gene sequencing analysis revealed eight different major phyla in the **saliva samples** (**Figure 3a**), dominated by Firmicutes (t0: mean 55.2%, median 56.8%; t1: mean 55.3%, median 50.5%; t2: mean 53.1%, median 55.5%), Actinobacteria (t0: 24.3%, 22.3%; t1: 24%, 26.4%; t2: 25.9%, 22.7%), Proteobacteria (t0: 9.5%, 7.5%; t1: 9.6%, 10.7%; t2: 9.5%, 10%), Bacteroidetes (t0: 7.4%, 6.4%; t1: 10%, 8.8%; t2: 7.3%, 8.6%), Fusobacteria (t0: 2.6%, 1.6%; t1: 3%, 2.4%; t2: 3.3%, 2.1%) as well as Campylobacterota, Candidatus Patescibacteria, and Cyanobacteria of low abundance. We also identified 15 major genera (**Figure 3b**), namely *Streptococcus* (t0: mean 36.7%, median 37.3%; t1: mean 33%, median 34.8%; t2: mean 34.9%, median 37.5%), *Rothia* (t0: 13.2%, 11.3%; t1: 10.2%, 11.4%; t2: 13.2%, 9.2%), *Veillonella* (t0: 10.2%, 11.2%; t1: 10.5%, 9.2%; t2: 9.5%, 7.7%), *Actinomyces* (t0: 9.3%, 8.2%; t1: 11%, 11.1%; t2: 10%, 8.9%), *Neisseria* (t0: 7.2%, 5.6%; t1: 7.2%, 7.2%; t2: 7.4%, 7.9%), *Prevotella* (t0: 5.5%, 4.6%; t1: 8.2%, 6.3%; t2: 5.8%, 6.7%), *Granulicatella* (t0: 2.9%, 2.8%; t1: 1.9%, 1.5%; t2: 2.3%, 2%), *Haemophilus* (t0: 1.9%, 1.9%; t1: 2%, 1.8%; t2: 1.8%, 1.9%), *Leptotrichia* (t0: 1.8%, 0.9%; t1: 2.2%, 2.3%; t2: 2.5%, 1%), *Gemella* (t0: 1.4%, 1.6%; t1: 1.2%, 1%; t2: 1.5%, 1.2%), *Fusobacterium* (t0: 0.8%, 0.7%; t1: 0.8%, 0.8%; t2: 0.8%, 0.6%), *Atopobium* (t0: 0.8%, 0.6%; t1: 1%, 1.2%; t2: 1.2%, 0.8%), *Porphyromonas* (t0: 0.7%, 0.5%; t1: 0.6%, 0.5%; t2: 0.7%, 0.7%), *Alloprevotella* (t0: 0.6%, 0.1%; t1: 0.7%, 0.1%; t2: 0.4%, 0.1%), and *Campylobacter* (t0: 0.6%, 0.5%; t1: 0.7%, 0.7%; t2: 0.6%, 0.6%).

The biofilm was composed of 12 different major phyla (**Figure 3c**). Here, the microbiome was dominated by Actinobacteria (t0: mean 42.6%, median 36.7%; t1: mean 37.4%, median 34.3%; t2: mean 45%, median 47%), Firmicutes (t0: 33.1%, 28%; t1: 32.4%, 26.3%; t2: 27.4%, 28.1%), Bacteroidetes (t0: 8.5%, 7.4%; t1: 12.8%, 12.1%; t2: 12.3%, 11.4%), Fusobacteria (t0: 7.3%, 8.5%; t1: 8.2%, 8%; t2: 8.9%, 7.6%), Proteobacteria (t0: 5.8%, 5%; t1: 5.6%, 2.2%; t2: 3.1%, 2%) as well as Campylobacterota, Candidatus Patescibacteria, Spirochaetes, Synergistetes, Desulfobacterota, Chlorobacteria, and Euryarchaeota (the latter from the domain of archaea) of low abundance. We again identified 15 major genera (**Figure 3d**): *Actinomyces* (t0: mean 20.6%, median 19.0%; t1: mean 19.4%, median 18.9%; t2: mean 23.8%, median 22.3%), *Streptococcus* (t0: 12.0%, 8.4%; t1: 10.2%, 8.5%; t2: 8.3%, 3.9%), *Veillonella* (t0: 6.9%, 4.5%; t1: 8.6%, 6.4%; t2: 4.5%, 2.5%), *Prevotella* (t0: 3.4%, 2.0%; t1: 6.4%, 5.9%; t2: 6.9%, 6.5%), *Rothia* (t0: 5.6%, 2.0%; t1: 5.1%, 1.4%; t2: 6.7%, 1.1%), *Leptotrichia* (t0: 4.5%, 2.6%; t1: 5.0%, 1.1%; t2: 5.6%, 2.6%), *Selenomonas* (t0: 4.3%, 2.7%; t1: 4.9%, 4.5%; t2: 7.5%, 7.2%), F0332 (*Actinomyces* oral taxon 848-like) (t0: 5.1%, 0%; t1: 4.4%, 0%; t2: 5.5%, 0%), *Corynebacterium* (t0: 7.1%, 5.9%; t1: 4.2%, 4.0%; t2: 5.6%, 4.8%), *Neisseria* (t0: 3.3%, 2.1%; t1:

3.3%, 1.4%; t2: 1.6%, 0.9%), *Fusobacterium* (t0: 2.8%, 2.5%; t1: 3.2%, 2.4%; t2: 3.4%, 2.5%), *Capnocytophaga* (t0: 3.3%, 3.4%; t1: 2.6%, 2.1%; t2: 1.4%, 1.1%), *Porphyromonas* (t0: 0.7%, 0.3%; t1: 2.3%, 0.4%; t2: 2.1%, 0.3%), *Bifidobacterium* (t0: 1.6%, 0.1%; t1: 2.0%, 0.1%; t2: 0.7%, 0.2%), and *Campylobacter* (t0: 1.7%, 1.3%; t1: 1.5%, 1.2%; t2: 1.3%, 1%).

The **fecal samples** presented 10 different major phyla (**Figure 3e**). Here, the microbiome was dominated by Firmicutes (t0: mean 71.5%, median 76.2%; t1: mean 71.2%, median 72.6%; t2: mean 67.6%, median 67%), Bacteroidetes (t0: 16.4%, 14.9%; t1: 19.8%, 16.6%; t2: 21.7%, 20.8%), Actinobacteria (t0: 8.2%, 2.5%; t1: 4.1%, 2.3%; t2: 6%, 2.4%), Verrucomicrobia (t0: 2.1%, 0.2%; t1: 2.5%, 0.2%; t2: 2.5%, 1.2%), Proteobacteria (t0: 1.2%, 1.1%; t1: 1.9%, 1.1%; t2: 1.8%, 1.1%) as well as Desulfobacterota, Cyanobacteria, Euryarchaeota, Fusobacteria, and Synergistetes of low abundance. The most abundant 15 genera (**Figure 3f**), were an unknown_*Lachnospiraceae* (t0: mean 10,8%, median 6,9%; t1: mean 9,9%, median 6,4%; t2: mean 7.4%, median 6.4%), *Blautia* (t0: 10.5%, 8.4%; t1: 12.6%, 11.9%; t2: 10.5%, 6.5%), *Faecalibacterium* (t0: 6.3%, 3,0%; t1: 7,8%, 7,3%; t2: 5,2%, 4,4%), *Ruminococcus* (t0: 5,6%, 5,0%; t1: 3,9%, 2,9%; t2: 4,7%, 5%), *Bifidobacterium* (t0: 5,3%, 1.4%; t1: 2.5%, 0.9%; t2: 3.7%, 0.8%), *Subdoligranulum* (t0: 4.3%, 3.6%; t1: 4%, 2.9%; t2: 1.7%, 0.9%), *Christensenellaceae* R-7 group (t0: 3.7%, 2.8%; t1: 1.4%, 0.2%; t2: 3.2%, 1.1%), *Alistipes* (t0: 2.4%, 1%; t1: 1.7%, 1.3%; t2: 2.8%, 1.7%), *Akkermansia* (t0: 2.1%, 0.2%; t1: 2.5%, 0.2%; t2: 2.5%, 1.2%), *Lachnospiraceae* NK4A136 group (t0: 2.1%, 1.3%; t1: 2.5%, 3.5%; t2: 1.5%, 1.5%), *Lachnospiraceae* UCG-002 (t0: 2.1%, 1.5%; t1: 1.3%, 1.5%; t2: 1.7%, 1.1%), *Anaerostipes* (t0: 2.1%, 1.3%; t1: 1.7%, 1.3%; t2: 1.9%, 1.8%), *Ruminococcus torques* group (t0: 2%, 1.2%; t1: 2%, 1.2%; t2: 2.3%, 1.9%), and *Collinsella* (t0: 1.9%, 0.7%; t1: 0.8%, 0%; t2: 1.4%, 0.6%).

IIIb) Supplementary microbiome data (patient level)

A number of six patients showed in total twelve individual changes in the microbiome

As outlined above, changes in the microbiome were mainly patient- and specimen-specific (with the most dynamic seen within biofilm, followed by fecal samples, and saliva). We therefore analyzed the most changes in certain specimens of shifters (in total **twelve events** in six patients) in more depth. Changes within twelve (**Figures 4a, c, e**) and twenty (**Figures 4b, d, f**) selected genera per specimen can be deduced from **Figure 4**. The overall significance of changes in certain taxa (all levels) is presented in **Figure 5** for comparison.

In patient **A01**, *Neisseria* were reduced in saliva and *Actinomyces*, *Capnocytophaga*, *Corynebacterium*, and *Neisseriaceae* in biofilm (the latter three significant over all biofilm samples, **Figure 5**). Anaerobes such as *Fusobacterium* and *Prevotella* increased, with the latter significantly over all saliva samples. In patient **A02** *Neisseriaceae*, *Capnocytophaga* (both significantly over all biofilm samples), and *Streptococcus* were reduced and *Actinomyces* increased in biofilm. In fecal samples of the same patient *Bifidobacterium* was reduced. *Subdoligranulum* almost disappeared and the *Ruminococcus torques* group increased her, both events found significantly over all fecal samples. In patient **A09** a deep loss of dominating bacteria was found in biofilm at t1, but - except for *Capnocytophaga* - most genera were recovered by t2. The microbiome of patient **A12** was found to be extraordinary in general, most likely because of a low-dosage antibiotic treatment which was not regarded high enough for exclusion from study. For instance, the A12-biofilm (**Figure 4c**) had low, decreasing amounts of *Actinomyces sensu lato* and almost no *Streptococcus*, *Neisseria* or *Capnocytophaga*. Instead, anaerobes such as *Veillonella*, *Prevotella* and *Fusobacterium* were over-represented and an otherwise not detected F0332-like *Actinomyces* clone dominating. In A12-saliva, a minor decrease of *Actinomyces* and increase of *Prevotella* was reflected. The fecal flora of this patient was not much extraordinary at baseline and week 1, however - after 4 weeks of SFOH administration - *Ruminococcus* (other than *R. torques* group), *Akkermansia*, *Alistipes*, and certain *Lachnospiraceae* were reduced, while *Christensenellaceae*, *Subdoligranulum* and especially *Bifidobacterium* increased (the latter two against the overall trend). In patient **A13** changes in saliva were again minor with decrease in *Actinomyces* and increase in anaerobic *Leptotrichia*. In contrast, certain *Blautia* (clone B2-11) first increased and then decreased in the fecal samples (following an overall significant shift, **Figure 5**). *Bacteroides*, *Faecalibacterium*, *Alistipes*, and certain *Lachnospiraceae* increased, while *Subdoligranulum* (significant according **Figure 5**) decreased. Finally, patient **A16** showed minor decrease in biofim-associated *Veillonella* (underlining significance in **Figure 5**) but major changes in the intestinal microbiome (increase of *Akkermansia* and *Blautia* on the cost of *Faecalibacterium*, *Bifidobacterium*, certain *Christensenellaceae*, and *Alistipes*).

References of Supplementary Information

1. Drakesmith H, Prentice AM. Hepcidin and the iron-infection axis. *Science* 2012; **338:** 768-772.

2. Alexeev D, Zhu H, Guo M*, et al.* A novel protein-mineral interface. *Nat Struct Biol* 2003; **10:** 297-302.

3. Braun V, Pramanik A, Gwinner T*, et al.* Sideromycins: tools and antibiotics. *Biometals* 2009; **22:** 3-13.

4. Butler A. Iron acquisition: straight up and on the rocks? *Nat Struct Biol* 2003; **10:** 240-241.
